# A polymorphism of the interleukin-1 **β** gene influences survival in pancreatic cancer

**DOI:** 10.1054/bjoc.2000.1479

**Published:** 2000-11-22

**Authors:** M D Barber, J J Powell, S F Lynch, K C H Fearon, J A Ross

**Affiliations:** University Department of Surgery, Royal Infirmary of Edinburgh, Edinburgh, EH3 9YW, Scotland, UK

**Keywords:** pancreatic cancer, interleukin-1β, genetic polymorphisms, prognostic factors, C-reactive protein

## Abstract

Pro-inflammatory cytokines contribute to the cachexia associated with pancreatic cancer and stimulate the acute phase response which has been associated with shortened survival in such patients. Polymorphisms of cytokine genes may influence their production. The present study examined the effect of a polymorphism of the interleukin (IL)-1b gene upon the inflammatory state and survival in pancreatic cancer. Genomic DNA was obtained from 64 patients with pancreatic cancer and 101 healthy controls. Using the polymerase chain reaction and subsequent TaqI restriction enzyme digestion the subject's genotype for a diallelic polymorphism of the interleukin-1b gene was established. IL-1b production by peripheral blood mononuclear cells and serum C-reactive protein (CRP) levels from patients were also examined and survival noted. Patients homozygous for allele 2 of the IL-1b gene had significantly shorter survival than other groups (P = 0.0001). These patients also exhibited higher IL-1b production (P = 0.022). Possession of allele 2 was also associated with significantly shorter survival (median 144 vs 256 days, P = 0.034) and significantly higher CRP level (P = 0.0003). The possession of a genotype resulting in increased IL-1b production was associated with shortened survival and increased serum CRP level. This may reflect the role of IL-1b in inducing an acute phase protein response and cachexia in cancer or may be related to changes in tumour phenotye. © 2000 Cancer Research Campaign http://www.bjcancer.com


*British Journal of Cancer* (2000) **83**(11), 1443–1447
© 2000 Cancer Research Campaign
doi: 10.1054/ bjoc.2000.1479, available online at http://www.idealibrary.com on IDEAL®

http://www.bjcancer.com

### A polymorphism of the interleukin-1β gene influences survival in pancreatic cancer

**MD Barber, JJ Powell, SF Lynch, KCH Fearon and JA Ross**

University Department of Surgery, Royal Infirmary of Edinburgh, Edinburgh EH3 9YW, Scotland, UK

Summary Pro-inflammatory cytokines contribute to the cachexia associated with pancreatic cancer and stimulate the acute phase response which has been associated with shortened survival in such patients. Polymorphisms of cytokine genes may influence their production. The present study examined the effect of a polymorphism of the interleukin (IL)-1b gene upon the inflammatory state and survival in pancreatic cancer. Genomic DNA was obtained from 64 patients with pancreatic cancer and 101 healthy controls. Using the polymerase chain reaction and subsequent TaqI restriction enzyme digestion the subject's genotype for a diallelic polymorphism of the interleukin-1b gene was established. IL-1b production by peripheral blood mononuclear cells and serum C-reactive protein (CRP) levels from patients were also examined and survival noted. Patients homozygous for allele 2 of the IL-1b gene had significantly shorter survival than other groups (P = 0.0001). These patients also exhibited higher IL-1b production (P = 0.022). Possession of allele 2 was also associated with significantly shorter survival (median 144 vs 256 days, P = 0.034) and significantly higher CRP level (P = 0.0003). The possession of a genotype resulting in increased IL-1b production was associated with shortened survival and increased serum CRP level. This may reflect the role of IL-1b in inducing an acute phase protein response and cachexia in cancer or may be related to changes in tumour phenotye. © 2000 Cancer Research Campaign http://www.bjcancer.com

**Keywords:** pancreatic cancer; interleukin-1β; genetic polymorphisms; prognostic factors; C-reactive protein

Despite progress in diagnosis and staging, pancreatic cancer still has a dismal prognosis (Ahlgren, 1996), however, it remains difficult to predict survival in patients with advanced pancreatic cancer. We have previously determined that nutritional and inflammatory factors such as albumin and C-reactive protein (CRP) are of major importance in determining survival (Falconer et al, 1995). This appears to be due to their relationship to the progressive nutritional decline or cachexia seen in the majority of patients with advanced pancreatic cancer, resulting in increased energy expenditure, reduced nutritional intake and death due to wasting (Warren, 1935; Falconer et al, 1994; Wigmore et al, 1997). However, albumin and CRP are sensitive to clinical events often experienced by patients around the time of diagnosis, such as jaundice, cholangitis and surgery, and thus they are of limited use in determining palliative therapy for these patients.

The acute phase protein response, of which the changes in albumin and CRP concentrations form a part, is modulated by pro-inflammatory cytokines. Many pro-inflammatory cytokines, including interleukin (IL)-1, IL-6, interferon-γ and tumour necrosis factor (TNF), will give rise to aspects of cachexia when administered in animal models (Mahony and Tisdale, 1988; Gelin et al, 1991; Hellerstein et al, 1989; Matthys et al, 1991; Strassmann et al, 1992). Although production of these cytokines depends on a variety of clinical factors, there is increasing evidence that genetic factors may be involved. Variations in the genes coding for IL-1 and TNF have been found to affect production of the relevant cytokines (Pociot et al, 1992; Stüber et al, 1996). If these polymorphisms were relevant to the survival of patients they would offer a window on the inflammatory state of the patient independent of their clinical condition and therefore may be useful in guiding palliative intervention. We have previously shown that two polymorphisms of the TNF gene do not influence the inflammatory state or survival in advanced pancreatic cancer (Barber et al, 1999).

This study examined a bi-allelic TaqI polymorphism of the IL-1β gene (13) in patients with advanced pancreatic cancer and related this to IL-1β production, CRP concentration and survival.

### METHODS

### Subjects

After informed consent, venous blood was collected for IL-1β genotyping from 64 consecutive patients with a diagnosis of unresectable pancreatic cancer based on histological or unequivocal radiological or operative findings. In order to assess the inflammatory state it was felt to be important to collect samples from patients without evidence of infection or jaundice approximately one month after diagnosis, surgery or endobiliary stenting. It was possible to obtain venous blood from 43 such patients for the measurement of serum CRP and 22 such patients for measurement of peripheral blood mononuclear cell (PBMC) IL-1β production.

Blood was also collected for IL-1β genotyping from 101 healthy volunteers from the same geographical area attending the Blood Transfusion Service Plasmapheresis Centre in Edinburgh.

*Received 11 May 2000*
*Revised 24 July 2000*
*Accepted 9 August 2000*

*Correspondence to:* JA Ross

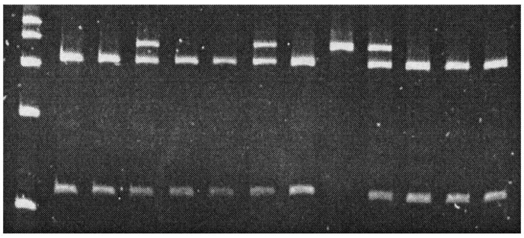

**Figure 1** 6% polyacrylamide gel showing interleukin-1β genotyping. 699 bp fragment of IL-1β gene has been amplified by PCR and digested with TaqI restriction enzyme. Allele 1 is cleaved into 175 and 524 bp fragments while allele 2 remains uncleaved at 699 bp

Survival was noted from time of histological confirmation of pancreatic adenocarcinoma (80%) or of unequivocal radiological or operative findings in those for whom histological confirmation was not obtained. Approval for the study was obtained from the Lothian Ethical Committee.

### IL-1β genotyping

DNA was extracted from 1 ml samples of EDTA anticoagulated blood using a Puregene DNA isolation kit based on a simple salting out technique (Miller et al, 1988) (Gentra systems, North Carolina, USA). The polymerase chain reaction (PCR) was used to amplify a 699 bp fragment of the IL-1β genomic sequence using primers upstream 5′ TGT TCT TAG CCA CCC CAC TC 3′ and downstream 5′ ATC GCT CCA GCA CTC TTG TT 3′ (Genosys, Pampisford, UK) designed on the basis of the published sequence (Clark et al, 1986) using the Primer 3 program (Rozen and Skaletsky, 1996). The following PCR protocol was used – 3 cycles of 97°C for 1.5 minutes, 54°C for 1.5 minutes, 74°C for 1 minute; 32 cycles of 97°C for 0.5 minutes, 54°C for 0.5 minutes, 74°C for 1.5 minutes; and 72°C for 10 minutes using reagents supplied by Promega (Southampton, UK) on a Hybaid Omn-E thermal cycler (Teddington, UK). The PCR product was digested directly with 0.5 μ of TaqI restriction enzyme (Appligene, Chester-le-Street, UK) at 65°C for 4 hours. Restriction enzyme products were analysed on 1% NuSieve agarose (FMC bioproducts, Rockland, Maine, USA) or 6% polyacrylamide gels (Biorad, Hemel Hempstead, UK). The cleaved product produced bands at 524 and 175 bp representing allele 1 while the uncleaved 699 bp product represented allele 2 (Figure 1).

### Cytokine production

Serum IL-1β levels were measured by ELISA (Quantikine High Sensitivity, R & D System, Abingdon, UK). Limit of detection was 0.2 pg $ml^{-1}$.

PBMCs were separated from 20 ml of heparin anticoagulated blood on a hypaque gradient (Histopaque 1077, Sigma, Poole, UK) by centrifuging at 1500 rpm for 30 minutes. Cells from the interface were removed and washed three times in cell culture medium (Roswell Park Memorial Institute medium 1640, Life Technologies, Paisley, UK) with penicillin, streptomycin and glutamine (2 mmol $l^{-1}$) (Sigma) added. Cells were resuspended, counted and cultured in 96-well, flat-bottomed tissue culture plates (Corning Coster, High Wycombe, UK) at a concentration of 2 × $10^5$ per well in 200 μl cell culture medium with 10% fetal calf serum in the presence of 10 μg $ml^{-1}$ *E. coli* lipopolysaccharide (Sigma). Supernatants from PBMC cultures were removed at 24 hours and stored at –70°C for subsequent analysis. IL-1β content of supernatants was measured by ELISA (Quantikine, R & D Systems, Abingdon, UK). Limit of detection was 4 pg $ml^{-1}$.

**Table 1** Characteristics of patients with advanced pancreatic cancer and healthy controls

| | | | Interleukin-1β genotype[a] | | |
|---|---|---|---|---|---|
| | **Age** | **Sex** | **1/1** | **1/2** | **2/2** |
| Cancer patients (*n* = 64) | 64<br>(57–72) | 30F<br>34 M | 68% | 25% | 6% |
| Healthy controls (*n* = 101) | 42<br>(36–47) | 32F<br>69 M | 55% | 40% | 5% |

M: male, F: female. [a]Comparison between groups by Chi squared test–*P* = 0.16.

Serum CRP levels were measured by an automated immunoturbidometric method (Abbott Laboratories, Maidenhead, UK). Limit of detection was 10 mg $l^{-1}$.

### Statistical analysis

Data are presented as median and interquartile range unless otherwise stated. Comparisons between groups of continuous variables were made by the Mann-Whitney U test. Categorical variables were compared by the Chi-squared test. Correlation between continuous variables were assessed by the Spearman rank correlation coefficient. Survival was analysed using the Kaplan-Meier technique and groups compared by the log rank test. A *P* value of less than 0.05 was taken to denote significance.

### RESULTS

Subject characteristics and prevalence of IL-1β genotypes are presented in Table 1. 30 patients had UICC stage II disease, 8 patients stage III and 26 patients stage IV. No subject underwent resectional surgery or received chemotherapy or radiotherapy. There was no significant difference in the distribution of genotypes between pancreatic cancer patients and healthy controls.

IL-1β was only detectable in serum from two patients (without infection or jaundice). One patient of genotype 2/2 had a serum level of 7.24 pg $ml^{-1}$ while a patient of 1/2 genotype had a level of 1.16 pg $ml^{-1}$. Both patients had substantially elevated serum CRP levels at 151 and 53 mg $l^{-1}$ respectively.

IL-1β production by PBMCs from cancer patients (without evidence of infection or jaundice, approximately 1 month after diagnosis, surgery or endobiliary stenting) by IL-1β genotype is presented in Figure 2. PBMC IL-1β production by those with a 2/2 genotype (median 9.7 ng $ml^{-1}$ (range 7.9–11.5)) was significantly higher than those with 1/2 (2.2 (interquartile range 1.5–2.7), $P = 0.046$) or 1/1 (1.9 (1.4–2.6), $P = 0.027$) genotype. There was no significant difference between PBMC IL-1β production between 1/2 and 1/1 genotype ($P = 0.72$). There was no significant relationship between PBMC IL-1β production and CRP level ($r = 0.28$, $P = 0.21$).

Serum CRP level by IL-1β genotype of cancer patients (without evidence of infection or jaundice, approximately 1 month after

© 2000 Cancer Research Campaign

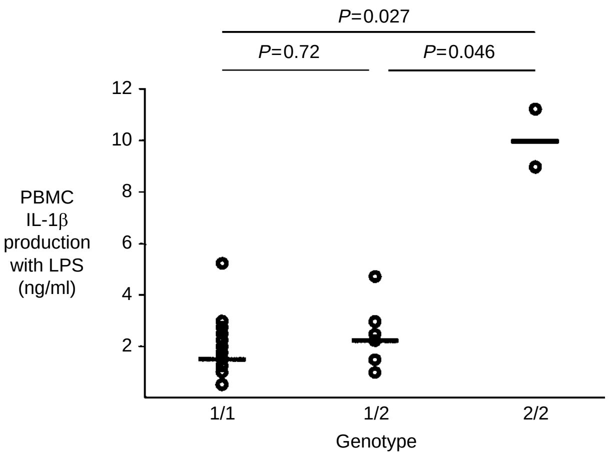

**Figure 2** Interleukin-1β production by peripheral blood mononuclear cells from 22 patients with advanced pancreatic cancer without infection or jaundice measured approximately 4 weeks after diagnosis, surgery or endobiliary stenting presented by IL-1β genotype (1/1 – 14 patients, 1/2 – 6 patients, 2/2 – 2 patients). Horizontal bar represents median value. Comparisons between groups by Mann-Whitney U test.

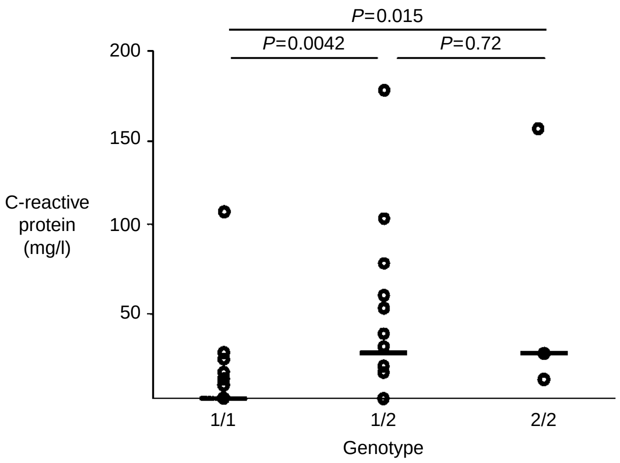

**Figure 3** Serum C-reactive protein levels from 43 patients with advanced pancreatic cancer without infection or jaundice measured approximately 4 weeks after diagnosis, surgery or endobiliary stenting presented by IL-1β genotype (1/1 – 28 patients, 1/2 – 12 patients, 2/2 – 3 patients). Horizontal bar represents median value. Comparisons between groups by Mann-Whitney U test.

diagnosis, surgery or endobiliary stenting) is presented in Figure 3. CRP levels from those with the 2/2 (28 mg $l^{-1}$ (20–130)) or 1/2 genotype (31 mg $l^{-1}$ (13–72)) were significantly higher than those with the 1/1 genotype (0 mg $l^{-1}$ (0–10)) (*P* = 0.015 and 0.0042, respectively). There was no significant difference between CRP levels from those with 2/2 and 1/2 genotypes (*P* = 0.42).

Survival of patients by IL-1β genotype is presented in Figure 4. Those with 2/2 genotype (88 days (69–108)) had significantly shorter survival than those with 1/2 (173 days (107–269)) or 1/1 (243 days (126–333)) genotype (*P* = 0.012 and 0.0001 respectively). The difference between survival of those with 1/1 and 1/2 genotype is not quite significant (*P* = 0.10). All 4 censored individuals possess the 1/1 genotype. Those possessing allele 2 had significantly shorter survival (148 days (85–245)) than those who did not (243 days (126–333)) (*P* = 0.043) (Figure 5).

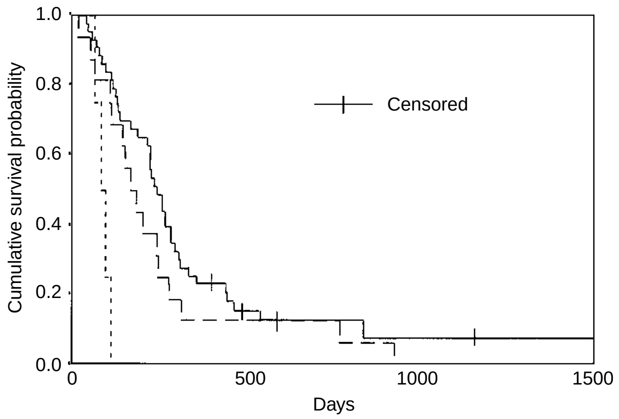

**Figure 4** Kaplan-Meier plot of survival of 64 patients with advanced pancreatic cancer presented by IL-1β genotype. Median survivals – 1/1 genotype 243 days (solid line), 1/2 173 days (broken line), 2/2 88 days (dotted line). Comparison by log rank test – overall *P* = 0.0005, 1/1 vs 1/2 *P* = 0.010, 1/1 vs 2/2 *P* = 0.0001, 1/2 vs 2/2 *P* = 0.012.

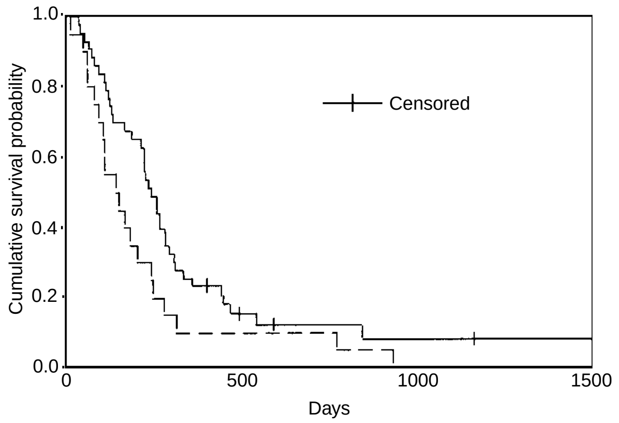

**Figure 5** Kaplan-Meier plot of survival of 64 patients with advanced pancreatic cancer stratified for possession of IL-1β allele 2. Median survivals – possession of allele 2–148 days (broken line), no allele 2–243 days (solid line). Comparison by log rank test – *P* = 0.043.

Disease stage was not a significant predictor of survival in this group of patients (*P* = 0.65). When survival by IL-1β genotype was split by disease stage genotype remained a significant predictor of survival in those with stage II and IV disease where sufficient numbers were available for analysis (*P* = 0.025 and *P* = 0.028, respectively).

For predicting survival at 6 months the possession of allele 2 has a sensitivity of 48%, a specificity of 81%, a positive predictive value of 65%, a negative predictive value of 67% and an accuracy of 67%.

### DISCUSSION

In this study of patients with advanced pancreatic cancer we have shown that IL-1β genotype appears to be important in determining IL-1β production, serum CRP level and survival from diagnosis.

There was no difference in the distribution of genotypes between normal subjects and cancer patients. The distribution found in normal subjects is very similar to that described in other

© 2000 Cancer Research Campaign

European populations (Pociot et al, 1992; Bioque et al, 1995; Heresbach et al, 1997). There was a trend towards fewer cancer patients possessing allele 2. If possession of allele 2 shortens survival we may have missed some patients who died before assessment, thus our estimate of the importance of allele 2 may be a conservative one.

It is rare to detect IL-1 in the serum of cancer patients, thus measures of production are more important for gauging in vivo levels (Moldawer and Copeland, 1997). Only one group has previously studied the relationship between this IL-1β polymorphism and production of IL-1β by leukocytes (Pociot et al, 1992). Examining a group of 45 healthy individuals they were able to show a stepwise increase in IL-1β production from 1/1 to 1/2 to 2/2 genotype. While numbers in the present study were insufficient to confirm this relationship, differences between the 2/2 genotype and the other groups were significant and there was a marked trend in agreement with this previous study ($P = 0.072$ – Kruskal-Wallis test).

The relationship between IL-1β genotype and the acute phase protein response has not previously been examined. It is known that the administration of IL-1α or β will induce an acute phase response in mice (Moldawer et al, 1988) and that the administration of antibodies to the IL-1 receptor will attenuate the acute phase response to turpentine abscess in mice (Gerschenwald et al, 1990). It is thought that the acute phase response is primarily activated by IL-6 (Castell et al, 1990) but it has been suggested that blockade of IL-1 action will reduce IL-6 production (Yasumoto et al, 1995). In the present study we failed to identify any simple relationship between serum CRP levels and IL-1β production although IL-1β genotype again seemed to be important with those possessing allele 2 having significantly higher CRP levels than those without.

The administration of pro-inflammatory cytokines, including IL-1, IL-6, interferon-α and TNF, produces features of cachexia in experimental models (Mahony and Tisdale, 1988; Hellerstein et al, 1989; Gelin et al, 1991; Matthys et al, 1991; Strassmann et al, 1992). These agents all induce an acute phase response and it has been suggested that the need for amino acids to manufacture acute phase reactants in the absence of adequate nutritional intake contributes to breakdown of skeletal muscle and so the weight loss of cachexia (Reeds et al, 1994). We have previously shown that the presence of an acute phase response is associated with nutritional decline and poor survival in pancreatic cancer (Falconer et al, 1994, 1995; Wigmore et al, 1997). In this study we have shown that IL-1β genotype is related to survival. The stepwise decrease in median survival between the 1/1, 1/2 and 2/2 genotypes would appear to correspond with the apparent increase in IL-1β production and would therefore be compatible with the hypothesis that a more marked pro-inflammatory state is detrimental to survival in pancreatic cancer, perhaps due to nutritional decline. It has also been suggested that pro-inflammatory cytokines will promote tumour growth in animal models (Gelin et al, 1991). Blockade of the IL-1 receptor has been shown to reduce liver metastasis in a melanoma model (Vidal-Vanaclocha et al, 1994) perhaps due to an IL-1 induced increase in endothelial receptor expression (Vidal-Vanaclocha et al, 1996).

The very poor survival of patients with 2/2 genotype is of limited value clinically due to the low prevalence of this genotype, however, the possession of allele 2 was also a significant predictor of survival. This was present in a third of the cancer patients in the present study and almost a half of the population as a whole so it may be a valuable clinical tool for predicting survival. It has been suggested that endoscopic palliation of jaundice rather than surgical biliary bypass is appropriate in those patients with a life expectancy of 6 months or less (van den Bosch et al, 1994; Rosewicz and Weidenmann, 1997). Existing measures of the inflammatory state are sensitive to surgery, infection and jaundice while IL-1β genotype is not. Using this method to predict 6-month survival had an accuracy of 67% in the current study suggesting that it may be useful in guiding palliative biliary drainage in these patients.

In conclusion, IL-1β genotype is related to IL-1β production and the level of the acute phase protein response and affects survival in patients with advanced pancreatic cancer.

### ACKNOWLEDGEMENTS

This work was supported in part by Scotia Pharmaceuticals, Ross Products Division, Abbott Laboratories and the University of Edinburgh. We thank Jean Maingay and Kathryn Sangster for their excellent technical assistance.

### REFERENCES

Ahlgren JD (1996) Epidemiology and risk factors in pancreatic cancer. *Semin Oncol* **23**: 241–250

Barber MD, Powell JJ, Lynch SF, Gough NJ, Fearon KCH and Ross JA (1999) Two polymorphisms of the tumour necrosis factor gene do not influence survival in pancreatic cancer. *Clin Exp Immunol* **117**: 425–429

Bioque G, Crusius JBA, Koutroubakis I, Bouma G, Kostense PJ, Meuwissen SGM and Peña AS (1995) Allelic polymorphism in IL-1β and IL-1 receptor antagonist (IL-1ra) genes in inflammatory bowel disease. *Clin Exp Immunol* **102**: 379–383

Castell JV, Gómez-Lechón MJ, David M, Fabra R, Trullenque R and Heinrich PC (1990) Acute-phase response of human hepatocytes: regulation of acute-phase protein synthesis by interleukin-6. *Hepatology* **12**: 1179–1186

Clark BD, Collins KL, Gandy MS, Webb AC and Auron PE (1996) Genomic sequence for human prointerleukin 1 beta: possible evolution from a reverse transcribed prointerleukin 1 alpha gene. *Nucleic Acids Res* **14**: 7897–7914

Falconer JS, Fearon KCH, Plester CE, Ross JA and Carter DC (1994) Cytokines, the acute-phase response, and resting energy expenditure in cachectic patients with pancreatic cancer. *Ann Surg* **219**: 325–331

Falconer JS, Fearon KCH, Ross JA, Elton R, Wigmore SJ, Garden OJ and Carter DC (1995) Acute-phase protein response and survival duration of patients with pancreatic cancer. *Cancer* **75**: 2077–2082

Gelin J, Moldawer LL, Lönnroth C, Sherry B, Chizzonite R and Lundholm K (1991) Role of endogenous tumor necrosis factor α and interleukin 1 for experimental tumor growth and the development of cancer cachexia. *Cancer Res* **51**: 415–421

Gerschenwald JE, Fong Y, Fahey TJ, Calvano SE, Chizzonite R, Kilian PL, Lowry SF and Moldawer LL (1990) Interleukin 1 receptor blockade attenuates the host inflammatory response. *Proc Natl Acad Sci USA* **87**: 4966–4970

Hellerstein MK, Meydani SN, Meydani M, Wu K and Dinarello CA (1989) Interleukin-1-induced anorexia in the rat. Influence of prostaglandins. *J Clin Invest* **84**: 228–235

Heresbach D, Alizadeh M, Dabadie A, Le Berre N, Colombel JF, Yaouanq J, Bretagne JF and Semana G (1997) Significance of interleukin-1β and interleukin-1 receptor antagonist genetic polymorphism in inflammatory bowel diseases. *Am J Gastroenterol* **92**: 1164–1169

Mahony SM and Tisdale MJ (1988) Induction of weight loss and metabolic alterations by human recombinant tumour necrosis factor. *Br J Cancer* **58**: 345–349

Matthys P, Dijkmans R, Proost P, van Damme J, Heremans H, Sobis H and Billiau A (1991) Severe cachexia in mice inoculated with interferon-γ-producing tumor cells. *Int J Cancer* **49**: 77–82

Miller SA, Dykes DD and Polesky HF (1988) A simple salting out procedure for extracting DNA fron human nucleated cells. *Nucleic Acids Res* **16**: 1215

Moldawer LL and Copeland EM (1997) Proinflammatory cytokines, nutritional support, and the cachexia syndrome. Interactions and therapeutic options. *Cancer* **79**: 1828–1839

*© 2000 Cancer Research Campaign*

Moldawer LL, Andersson C, Gelin J and Lundholm KG (1988) Regulation of food intake and hepatic protein synthesis by recombinant-derived cytokines. *Am J Physiol* **254**: G450–456

Pociot F, Mølvig J, Wogensen L, Worsaae H and Nerup J (1992) A TaqI polymorphism in the human interleukin-1β (IL-1β) gene correlates with IL-1β secretion *in vitro*. *Eur J Clin Invest* **22**: 396–402

Reeds PJ, Fjeld CR and Jahoor F (1994) Do the differences between the amino acid compositions of acute-phase and muscle proteins have a bearing on nitrogen loss in traumatic states? *J Nutr* **124**: 906–910

Rosewicz S and Weidenmann B (1997) Pancreatic carcinoma. *Lancet* **349**: 485–489

Rozen S and Skaletsky HJ (1996) Primer 3. http://www.genome.wi.mit.edu/genome_software/other/primer3.html

Strassmann G, Fong M, Kenney JS and Jacob CO (1992) Evidence for the involvement of interleukin 6 in experimental cancer cachexia. *J Clin Invest* **89**: 1681–1684

Stüber F, Petersen M, BokeImann F and Schade U (1996) A genomic polymorphism within the tumor necrosis factor locus influences plasma tumor necrosis factor-a concentrations and outcome of patients with severe sepsis. *Crit Care Med* **24**: 381–384

van den Bosch RP, van der Schelling GP, Klinkenbijl JHG, Mulder PGH, van Blankenstein M and Jeekel J (1994) Guidelines for the application of surgery and endoprostheses in the palliation of obstructive jaundice in advanced cancer of the pancreas. *Ann Surg* **219**: 18–24

Vidal-Vanaclocha F, Amézaga C, Asumendi A, Kaplanski G and Dinarello CA (1994) Interleukin-1 receptor blockade reduces the number and size of murine B16 melanoma hepatic matastases. *Cancer Res* **54**: 2667–2672

Vidal-Vanaclocha F, Alvarez A, Asumendi A, Urcelay B, Tonino P and Dinarello CA (1996) Interleukin 1 (IL-1)-dependent melanoma hepatic metastasis in vivo; increased endothelial adherence by IL-1-induced mannose receptors and growth factor production in vitro. *J Natl Cancer Inst* **88**: 198–205

Warren S (1935) The immediate causes of death in cancer. *Am J Med Sci* **184**: 610–616

Wigmore SJ, Plester CE, Ross JA and Fearon KCH (1997) Contribution of anorexia and hypermetabolism to weight loss in anicteric patients with pancreatic cancer. *Br J Surg* **84**: 196–197

Yasumoto K, Mukaida N, Harada A, Kuno K, Akiyama M, Nakashima E, Fujioka N, Mai M, Kasahara T, Fujimoto-Ouchi K, Mori K, Tanaka Y and Matsushima K (1995) Molecular analysis of the cytokine network involved in cachexia in colon 26 adenocarcinoma-bearing mice. *Cancer Res* **55**: 921–927

© 2000 Cancer Research Campaign
